# A pan-tumor-siRNA aptamer chimera to block nonsense-mediated mRNA decay inflames and suppresses tumor progression

**DOI:** 10.1016/j.omtn.2022.07.017

**Published:** 2022-07-20

**Authors:** Daniel Meraviglia-Crivelli, Helena Villanueva, Ashwathi Puravankara Menon, Angelina Zheleva, Beatriz Moreno, María Villalba-Esparza, Fernando Pastor

**Affiliations:** 1 Molecular Therapeutics Program, Center for Applied Medical Research, CIMA, University of Navarra, Pamplona 31008, Spain; 2 Instituto de Investigación Sanitaria de Navarra (IDISNA), Recinto de Complejo Hospitalario de Navarra, Pamplona 31008, Spain; 3 Department of Pathology, Yale University School of Medicine, New Haven, CT 06510, USA

**Keywords:** oligonucleotides: therapies and applications, aptamer, cancer immunotherapy, RNAi delivery, NMD

## Abstract

Immune-checkpoint blockade (ICB) therapy has changed the clinical outcome of many types of aggressive tumors, but there still remain many cancer patients that do not respond to these treatments. There is an unmet need to develop a feasible clinical therapeutic platform to increase the rate of response to ICB. Here we use a previously described clinically tested aptamer (AS1411) conjugated with SMG1 RNAi (AS1411-SMG1 aptamer-linked siRNA chimeras [AsiCs]) to inhibit the nonsense-mediated RNA decay pathway inducing tumor inflammation and improving response to ICB. The aptamer AS1411 shows binding to numerous mouse and human tumor cell lines tested. AS1411 induces tumor cytotoxicity in long incubation times, which allows for the use of the aptamer as a carrier to target the RNAi inhibition to the tumor. The AS1411-SMG1 AsiCs induce a strong antitumor response in local and systemic treatment in different types of tumors. Finally, AS1411-SMG1 AsiCs are well tolerated with no detected side effects.

## Graphical abstract

**Figure:**
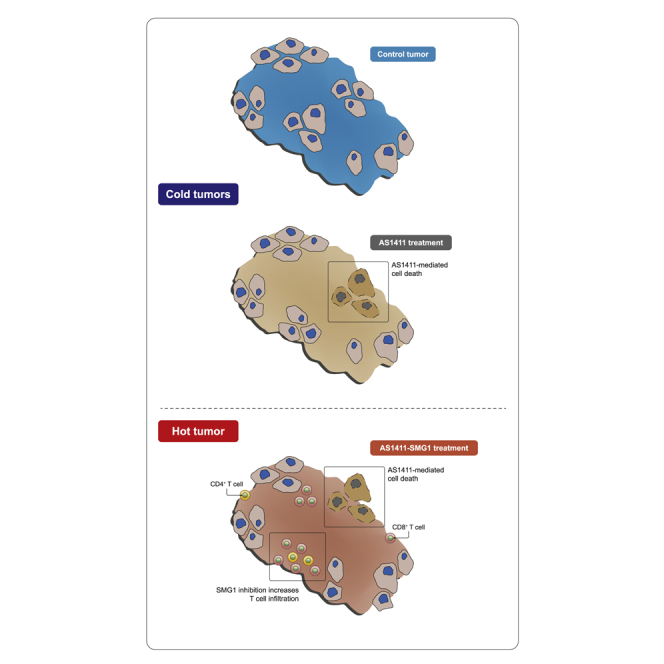

## Teaser

A1411 aptamer with a broad spectrum of tumor recognition is used to target SMG1 inhibition via siRNA delivery in different tumor models. This approach promotes a potent antitumor response in association with high immune infiltration in the tumor milieu.

## Introduction

Cancer immunotherapy has revolutionized oncology treatment with impressive results in a set of cancer patients. Immune-checkpoint blockade (ICB) therapy with anti-PD(L)-1 and anti-CTLA-4^1^^,^^2^ antibodies, along with CAR-T and adoptive cell therapies, have spearheaded the advances in cancer immunotherapy with outstanding responses.^3^^,^^4^ Despite these advances in the field, a large fraction of patients do not respond to cancer immunotherapy. One important limitation, among many others, that could explain the limited therapeutic effect of ICB on these patients might be the limited antigenicity of tumors. Antigenicity is conditioned by the source and quality of neoantigens expressed in each tumor. This is a serendipity process acquired by the tumor during its ontogenicity as it accumulates somatic mutations. Tumors with higher mutation burden usually are more likely to respond to ICB therapies.^5^, ^6^, ^7^, ^8^ The mutational load (mutanome) is an intrinsic fingerprint unique to each individual tumor lesion. Therefore, approaches to enhance antigenicity through increasing the mutation rate of tumors are technically cumbersome and risky.^9^, ^10^, ^11^ Another possible way to enhance tumor antigenicity is by inducing the expression of novel genome encoded transcripts that usually remain cryptic. For example, epigenetic drugs or splicing modulatory drugs can induce the expression of cryptic universal antigens such as testis antigens^12^ or aberrant splicing variants.^13^ Another strategy may involve blocking the nonsense-mediated mRNA decay (NMD)^14^,^51^. NMD is an RNA surveillance process that eliminates mRNA containing premature stop codons (PTC) that mainly appear during transcription by RNA polymerase errors or during RNA maturation. Furthermore, NMD inhibition has been shown to induce profound changes in the transcriptome including the expression mRNA that not necessarily contain PTC.^15^ Besides, many potent tumor acquired mutations originated by indels or gene translocations can lead to frameshift mutations frequently eliminated by NMD.^16^^,^^17^ Therefore, NMD disruption may not only lead to the expression of universal cryptic neoantigens, but also cryptic private tumor neoantigens.

Epigenetic drugs such as DNMT inhibitors (e.g., 5-aza-2-deoxycytidine [5-AZA]) promote enhanced antitumor responses when combine with ICB.^18^ Similarly, splicing modulator drugs (e.g., FDA approved because of other antitumor effects) have also shown augmented responses to ICB in preclinical settings.^13^ Even though there is still no clinically translational approved drug specific for NMD inhibition, the epigenetic modulator 5-AZA may exert part of its function as an indirect inhibition of NMD activity^19^ showing stabilization of indel-derived antigens that are under NMD control.^20^ Tumor-targeting aptamer-linked siRNA chimeras (AsiCs)^21^, ^22^, ^23^ are an alternative to small molecule inhibitor drugs with the advantage of making a chosen target gene druggable in a subset of target cells, minimizing potential undesirable side effects and therefore amplifying the therapeutic window. Aptamers are single-stranded oligonucleotide ligands that bind to their target with high affinity. As they are oligonucleotides, they can be chemically synthesized for mass production and attached to other oligonucleotide cargos such as siRNA. Herein, we use the DNA G4 quadruplex structure AS1411 aptamer that is presumed to bind to nucleolin to deliver NMD inhibition to the tumor site. The aptamer was discovered serendipitously in the 1990s as an oligonucleotide with tumor cytotoxic effects and was catapulted to the clinical arena in different trials^24^^,^^25^; but it was abandoned with modest antitumor efficacy.^26^ This aptamer can be rescued as a powerful therapeutic agent for cancer immunotherapy conserving the cytotoxic effect while efficiently delivering NMD inhibition to induce inflammation in the tumor milieu. Eli Gilboa’s team has pioneered the use of AS1411 to enhance tumor immunity by delivering RNAi to tumors disrupting TAP^27^ and also NMD.^28^ Herein we extend the characterization of this aptamer RNAi to inhibit NMD as a tool in the cancer immunotherapy arsenal. The aptamer binds to a plethora of human and mouse solid tumor cell lines, making it a broadly applicable drug. The injection of AS1411-SMG1 AsiC shows a potent antitumor response in tumor models in association with high immune cell infiltration. Furthermore, the treatment with AS1411-SMG1 AsiCs sensitizes the tumor to ICB therapy with anti-CTLA-4/PD-1 antibodies.

## Results

### AS1411 binds to different murine and human tumor cell lines and elicits cell cytotoxic effect

**Figure 1:**
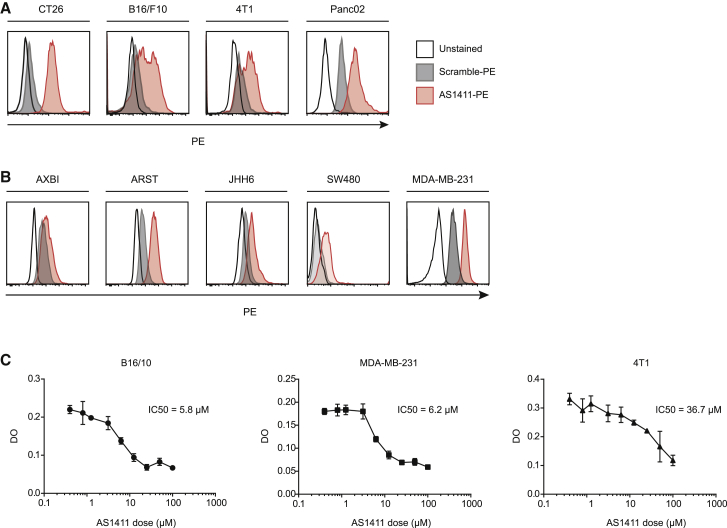
AS1411 binds and triggers tumor cytotoxicity in human and murine tumor cells (A) AS1411 aptamer binding to murine cancer cell lines of colon carcinoma (CT26), melanoma (B16/F10), breast cancer (4T1), and pancreatic cancer (Panc02). (B) AS1411 binds to human tumor cells: melanoma (AXBI, ARST), hepatocellular carcinoma (JHH6), colorectal adenocarcinoma (SW480), and breast cancer (MDA-MB-231). (C) Cytotoxic effect of AS1411 measured by MTS in human and mouse tumor cell lines. IC_50_ is indicated for each cell line. n = 3. Data shown are mean ± SEM.

The antitumor effect of AS1411 was initially described in human tumor cell lines. Firstly, we tested if the aptamer could cross-react with murine tumor cell lines. To that end, we labeled the AS1411 aptamer with biotin and used streptavidin-PE to track the binding of the aptamer to different tumor cell lines (Figures 1A and 1B). The range of binding varies among cell lines but is considerably high in most of them, independently of their human or murine origin.

The next question was to determine the cytotoxic effect mediated by AS1411 in human and mouse tumor cells. We used the murine melanoma cell line (B16/F10), the human breast cancer cell (MDA-MB-231), and the murine breast cancer cell line (4T1) and evaluated cell growth by MTS in the presence of increasing concentration of AS1411. The IC_50_ of the aptamer for B16/F10 was 5.8 μM, for MDA-MB-231 was 6.2 μM, and for 4T1 was 36.7 μM (Figure 1C). Interestingly, we observed that the aptamer exerts its main cytotoxic effect many days after the addition of the aptamer (with optimal effect at day 6). This long-time frame of action before the cell line dies opens the possibility to intervene and modulate the NMD pathway by delivering a siRNA, which usually has its full inhibitor effect 24–72 h after administration.

### AS1411-SMG1 AsiCs reduce SMG1 expression unmasking NMD-control neoantigens

**Figure 2:**
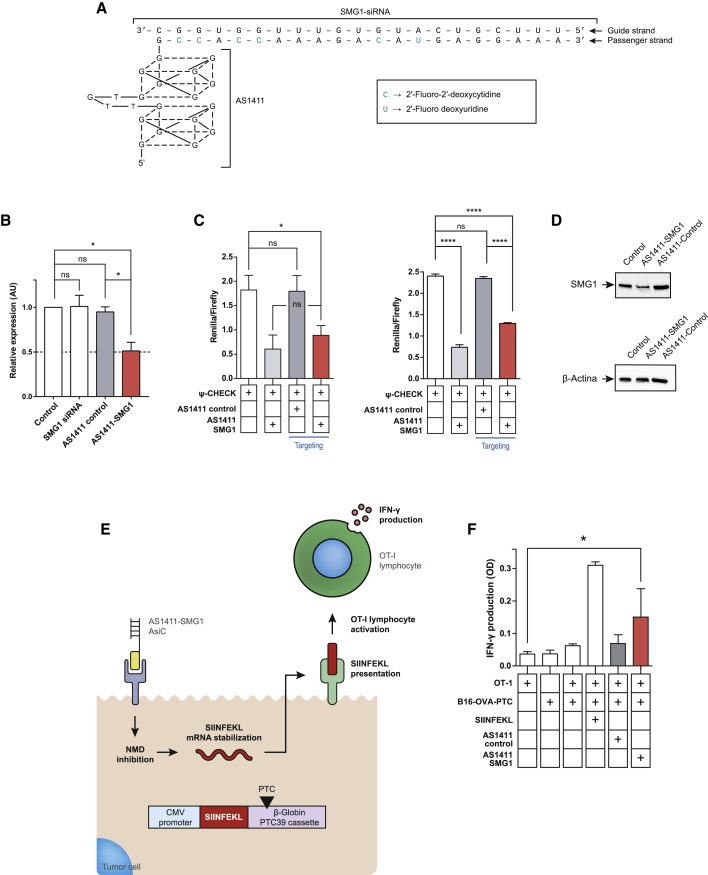
AS1411-siRNA SMG1 AsiC downregulates NMD via SMG1 silencing in tumor cells and improves the stability of potential NMD-regulated neoantigens (A) Schematic of AS1411-siRNA SMG1 AsiC. AS1411 is shown as G4 monomer conformation. Thymidines within the G4 are not represented for graphical simplification. 2′-Fluoro-modified nucleotides are indicated in green, and siRNA guide and passenger strands are specified with arrows. (B) AS1411-SMG1 AsiCs inhibit SMG1 mRNA by free uptake in CT26 cells. AS1411-AsiC was added twice at 24 and 48 h, and SMG1 mRNA was quantified by qRT-PCR. n = 3. (C) psiCHECK reporter assay to validate target inhibition of SMG1 in left, Panc02 cells and right, B16/F10. Cells transfected with psiCHECK luciferase reported plasmid containing the SMG1 target were treated as in (B) with AS1411-AsiC. SMG1 downregulation was proportional to the Renilla signal and normalized with Firefly luciferase. n = 3. (D) Western blot to validate AS1411 AsiC SMG1 silencing by free uptake. Treatment schedule followed was the same as in (B). (E) AS1411-SMG1 free uptake inhibits NMD in SIIN-BG-ter-expressing B16/F10 cells. This stabilizes SIINFEKL mRNA and leads to peptide presentation triggering OT-I lymphocytes activation. (F) B16/F10 expressing a β-Globin-SIINFEKL-PTC39 plasmid were treated with AS1411-SMG1 AsiC as in Figure 2C and then co-cultured with OT-I splenocytes. Supernatants were analyzed by IFN-γ ELISA. n = 3. Data shown are mean ± SEM. p < 0.05(^∗^), p < 0.01(^∗∗^), p < 0.001(^∗∗∗^), and p < 0.0001 (^∗∗∗∗^).

The aptamer core is of DNA and at the 3′ end was elongated with the RNA sequence containing 2′-fluoro-modified pyrimidines of the passenger strand specific for SMG1 NMD factor; the aptamer was further hybridized with the guide strand with no modifications (Figure 2A). AsiC hybridization quality was confirmed in each batch by PAGE (Figure S1A). AS1411-SMG1 AsiCs (AS1411 aptamer conjugated with SMG1 siRNA) binding to murine cells was confirmed by flow cytometry in B16/F10 cell line (Figure S1B). As negative control, we used a Scramble-SMG1 AsiC (Scramble aptamer conjugated with SMG1 siRNA) to check that the binding was AS1411 dependent (Figure S1B).

To confirm SMG1 silencing induced by the AS1411-SMG1 AsiC SMG1, we transfected it into murine colon cancer cell line CT26 (Figure S1C) or added directly on the cells for free uptake (Figure 2B). As negative control, we employed an AS1411-control AsiC (AS1411 aptamer conjugated with control siRNA) (Figure 2B) and Scramble-SMG1 AsiC to assess that the targeting to the cancer cells was AS1411 dependent (Figure S1D). Three days later, SMG1 mRNA levels were measured by qRT-PCR (Figures 2B, S1C, and S1D). To confirm these results, we also used a psiCHECK luciferase reporter vector, in which the SMG1 siRNA target was cloned downstream of the Renilla luciferase gene. The vector also contained the Firefly luciferase gene for signal normalization. Targeting inhibition of SMG1 was assessed as well in Panc02 and B16/F10 tumor cells pre-transfected with SMG1-psiCHECK reporter luciferase system, and we observed a significant reduction of luciferase signal proportional to the level of RNAi-mediated inhibition in both cell lines (Figure 2C). Next, we wanted to determine if this reduction in SMG1 target was sufficient to impact NMD activity and increase the stability of potential antigens that can be eliminated by NMD. To address this, we used a BG-ter plasmid modified to express SIINFEKEL (antigen peptide) downstream of the PTC (SIIN-BG-ter), whose stabilization will lead to a higher level of SIINFEKEL peptide specifically recognized by OT-I lymphocytes (Figure 2E). We measured the IFN-γ production of OT-I lymphocytes co-cultured with syngeneic tumor cells (B16/F10) expressing SIIN-BG-ter and pretreated with AS1411 control or SMG1 AsiC (Figure 2F). The highest levels of IFN-γ production by OT-I cells were achieved when the tumor was pretreated with AS1411-SMG1 AsiC (Figure 2F).

### Intratumoral injection of AS1411-SMG1 AsiC elicits a strong antitumor response

**Figure 3:**
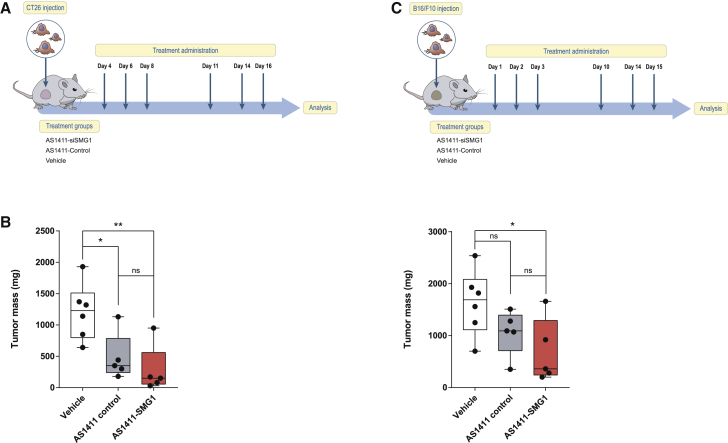
Intratumoral administration of AS1411-SMG1 AsiC significantly reduces tumor growth (A) Treatment schedule. CT26 cells were implanted into the right flank of Balb/c mice and AS1411-AsiC was injected intratumorally on days 4, 6, 8, 11, 14, and 16. Tumors were resected at day 20. (B) CT26 tumors were resected and weighed on day 20. n = 5–6. (C) Treatment schedule. B16/F10 cells were implanted into the right flank of C57/BL6 mice. AS1411-AsiC was injected intratumorally on days 1, 2, 3, 10, 14, and 15. On day 16, tumors were resected. (D) B16/F10 tumor weight on day 16. n = 5–6. Data shown are mean ± SEM. p < 0.05(^∗^), p < 0.01(^∗∗^), p < 0.001(^∗∗∗^), and p < 0.0001 (^∗∗∗∗^).

In order to test whether the AS1411-SMG1 AsiC can be used as an antitumor therapeutic agent, we first administered AS1411-SMG1 AsiC as a local injection in tumor-bearing mice. We evaluated the efficacy in two different tumor models: CT26 colon carcinoma and B16/F10 melanoma. We started the study with the less aggressive CT26 colon carcinoma model subcutaneously in Balb/c mice. AS11411-SMG1 AsiC was injected intratumorally on days 4, 6, 8, 11, 14, and 16 (Figure 3A), and on day 20 the mice were sacrificed to weigh the tumor mass. The treatment in the CT26 model exerts a strong antitumor effect with AS1411-control AsiC possible as a direct cytotoxic effect of AS1411; however the addition of SMG1 RNAi seems to further boost the antitumor response, increasing the grade of significance compared with the untreated group (Figure 3B). In B16/F10 C57/BL6 melanoma model, we initiated the treatment early following the schedule depicted in Figure 3C. Despite the aggressiveness of the B16/F10 model the antitumor response mediated by the cytotoxic effect of AS1411 aptamer was still quite strong with significant impact on tumor growth of mice treated locally with either AS1411-control AsiC or AS1411-SMG1 AsiC (Figure 3D). Nonetheless, the mice treated with AS1411-SMG1 AsiC seem to have smaller tumor mass compared with the untreated group (Figure 3D).

### AS1411-SMG1 AsiC injection induces high CD8 lymphocyte infiltration

**Figure 4:**
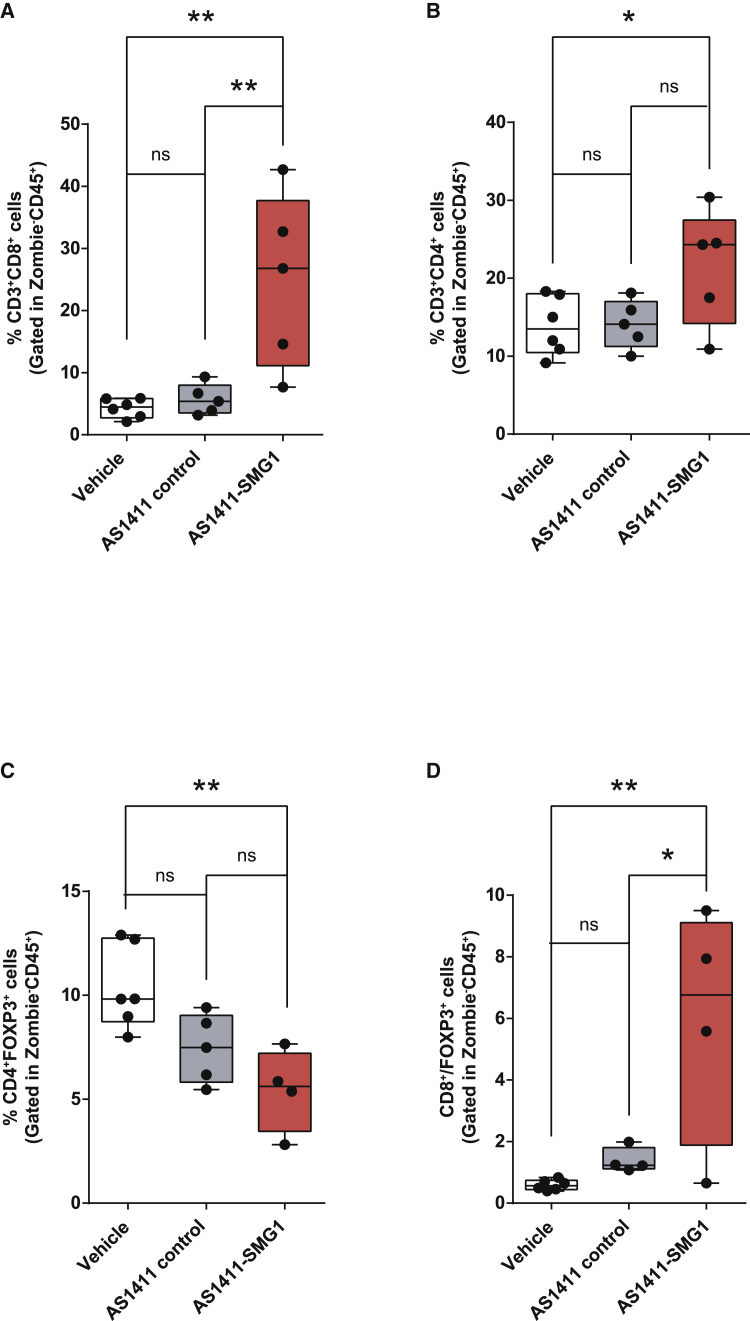
T cell infiltration increases in the B16/F10 tumor model after AS1411-SMG1 AsiC treatment. B16/F10 cells were injected into the right flank of C57/BL6 mice Mice were treated with six doses (300 pmol per dose) of AS1411-SMG1, AS1411-control, or vehicle (see Figure 3D for detailed treatment schedule). Tumors were resected on day 16 to analyze the lymphocyte infiltrate by flow cytometry. T cell populations were gated in the CD45^+^ Live cells (Zombie Green negative population) and quantified in percentage. (A) CD3^+^CD8^+^. (B) quantification of CD3^+^CD4^+^ T cells. (C) T regulatory lymphocytes (CD4^+^FOXP3^+^). (D) CD8^+^: T regulatory cells coefficient. n = 4–6. Data shown are mean ± SEM. p < 0.05(^∗^), p < 0.01(^∗∗^), p < 0.001(^∗∗∗^), and p < 0.0001 (^∗∗∗∗^).

To confirm if the possible improved outcome in mice treated with the AS1411-SMG1 AsiC was dependent on the immune response, we performed an immune cell infiltrate study using flow cytometry. Tumor cells obtained after the treatment schedule (Figure 3D) were harvested, mechanically and enzymatically digested, and stained with anti-CD3, anti-CD8, anti-CD4, and anti-FOXP3 antibodies. The frequency of the most important T lymphocyte subsets in the tumor milieu was assessed by flow cytometry (Figure 4). We observed that the treatment with AS1411-SMG1 AsiC and not with AS1411-control AsiC elicits a profound change in the tumor immune microenvironment with a high infiltration of CD8 cytotoxic lymphocytes (Figure 4A), a slight increase of CD4 helper lymphocytes (Figure 4B), and a reduction of Foxp3 Treg lymphocytes (Figure 4C), and the ratio of CD8/Foxp3 lymphocytes was augmented (Figure 4D). Higher infiltration of T lymphocytes was also confirmed in the CT26 tumor model via immunohistochemistry analysis (Figure S2). These results prove that NMD inhibition by AS1411-SMG1 AsiC is required to promote tumor inflammation as the AS1411-control AsiC is not triggering any increase in the tumor immune infiltration (Figures 4 and S2).

### Systemic AS1411-SMG1 AsiC injection elicits an antitumor response, further enhanced by CTLA-4/PD-1 blockade

Tumor disseminated lesions in some cancer patients might not be accessible or even undetected. Thus, evaluating the effect of the AS1411-SMG1 AsiC to target distal tumors is desirable as a more clinically feasible intervention. After observing the potent antitumor response induced by intratumoral treatment with AS1411-SMG1 AsiC, we tested its therapeutic efficacy upon systemic administration, distal to the tumor site following a different schedule of treatment.

**Figure 5:**
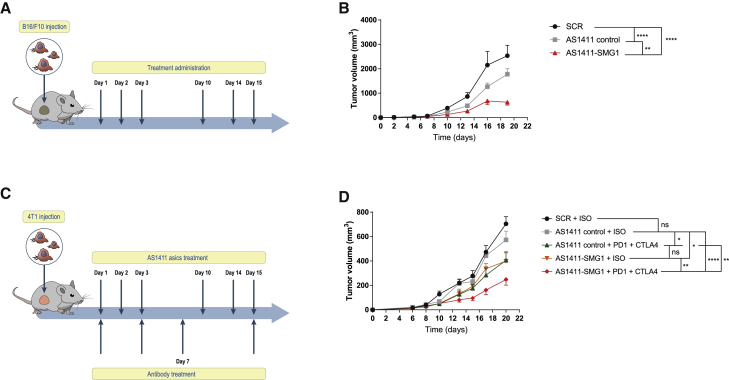
Antitumor effect of AS1411-SMG1 AsiC administered systemically (A) Treatment schedule. B16/F10 cells were implanted in C57/BL6 mice. 300 pmol of AS1411 AsiCs or scramble aptamer were administered intravenously on the indicated days. (B) AS1411-SMG1 treatment showed a highly significant antitumor effect compared with controls. n = 7–10. (C) Treatment schedule. 4T1 cells were injected in Balb/c mice. Mice were injected with CTLA-4 and PD-1 (100 μg of each antibody or 200 μg of isotype control antibody) on the days shown. (D) Additive effect of CTLA-4 + PD-1 checkpoint blockade in 4T1 murine breast cancer model. NMD inhibition with chimera showed a similar effect with the immune-checkpoint blockade therapy. AS1411-control per se had almost no significant effect. A significant improvement in antitumor effect was observed in the combination of AS1411-SMG1 CTLA-4 + PD-1 compared to both monotherapies. n = 6–10. Data shown are mean ± SEM. p < 0.05(^∗^), p < 0.01(^∗∗^), p < 0.001(^∗∗∗^), and p < 0.0001 (^∗∗∗∗^).

We treated B16/F10 melanoma tumor-bearing mice via systemic injection of AS1411-SMG1 AsiC, and tumor growth was monitored (Figure 5A). Mirroring the effect previously observed in B16/F10 tumor-bearing mice treated intratumorally with AS1411-SMG1 AsiC, systemic administration of the AS1411-control AsiC in B16/F10 the AS141-control AsiC again showed a significant reduction in tumor progression compared with untreated mice. This antitumor effect was enhanced in B16/F10 tumor-bearing mice upon systemic administration of the AS1411 SMG1 AsiC (Figure 5B).

We hypothesized that the induction of tumor inflammation with the treatment of AS1411-SMG1-AsiC creates the optimal conditions to sensitize the tumor to the action of ICB agents such as anti-CTLA-4/PD-1 antibodies. To evaluate this possibility, we used a breast cancer model that remains quite refractory to ICB therapy. 4T1 breast cancer-bearing mice were treated systemically with AS1411-SMG1 AsiC in combination with anti-CTLA-4/PD-1 antibodies (Figure 5C). Anti-CTLA-4/PD-1 treatment under this schedule induced a partial improvement in the tumor progression, similar to the observed with AS1411-SMG1 AsiC treatment. Mice with better antitumor responses were those treated with the full combination: anti-CTLA-4/PD-1 antibodies and AS1411-SMG1 AsiC (Figure 5D). Interestingly, the response to AS141-control was negligible, confirming that this type of tumor may be more resistant to the cytotoxic effect of AS1411 (Figure 1C).

### AS1411-SMG1 AsiC treatment has no major immune-related side effects

**Figure 6:**
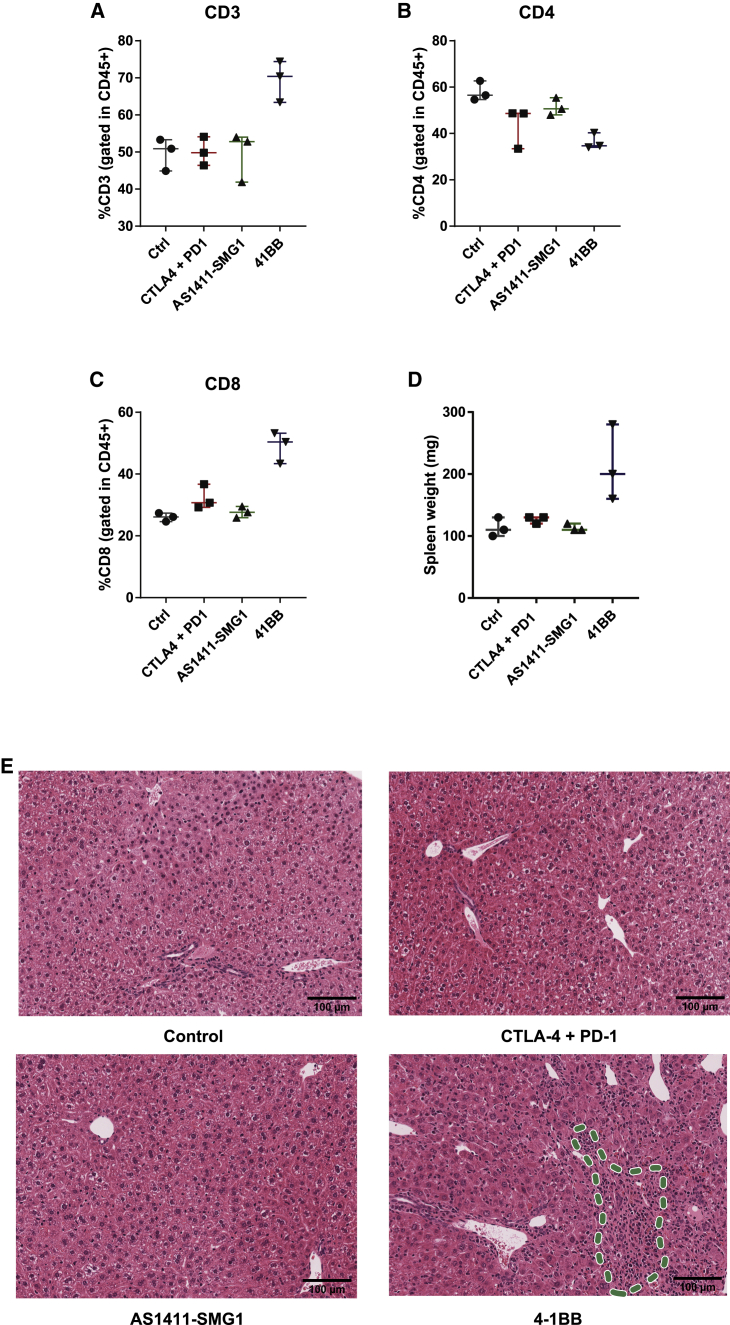
Evaluation of immune-mediated side effects of AS1411-SMG1 AsiC in mice Tumor-free C57/BL6 mice were treated with therapeutic doses used in Figure 5C of AS1411-SMG1 AsiC, anti-CTLA-4 (clone 9H10), and anti-PD-1 (clone rmp1-14) antibodies or three doses of 200 μg of anti 4-1BB antibody (clone 3H3) as control immune-mediated toxicity. n = 3. (A–C) Liver samples were analyzed by flow cytometry to study toxicity-induced inflammation. Total CD3^+^ (A) were quantified in the whole immune population (CD45^+^). CD4 (B) T cells were analyzed in the CD3^+^ cluster as well as CD8 lymphocytes (C). (D) Evidence of splenomegaly as side effect of systemic inflammation. (E) Hematoxylin and eosin staining of liver samples of treated mice. Abnormal infiltration of lymphocytes was detected in the samples of 4-1BB-treated mice as expected (indicated with dashed lines in the bottom right panel). No other major histological changes were observed. Data shown are mean ± SEM.

The high levels of tumor lymphocyte infiltration observed with the treatment of AS1411-SMG1 AsiC raises the concern of potential side effects in healthy non-targeted tissues, especially when the aptamer is injected systemically. To address this concern, we conducted a toxicity analysis on mice treated with a therapeutic dose of AS1411-SMG1 AsiC, following the same schedule (Figure 5C). In parallel, mice were also injected with anti-CTLA-4/PD-1 antibodies on the same schedule shown in Figure 5C. Of note, the AS1411-SMG1 AsiC and the anti-CTLA-4/PD-1 antibodies display similar antitumor effect in the 4T1 tumor model (Figure 5D). Thus, we are comparing toxicity induced by two different treatments with similar therapeutic outcomes. As a positive control, we used the 4-1BB agonistic antibody (3H3), which is a therapeutic agent that also induces high levels of lymphocyte infiltration in the tumor while triggering systematic and hepatic toxicity. Characterized by its propensity for high unspecific uptake, the liver is the organ most likely to be affected by therapeutic drugs, including oligonucleotide-based therapy,^29^ as an organ with high unspecific uptake of oligonucleotide-based molecules.^30^^,^^31^ To assess the extent of liver inflammation induced by each treatment, we performed flow cytometry analysis of CD8+ and CD4+ lymphocytes that infiltrate the liver. We observed clearly that the 4-1BB agonistic antibody, as previously reported, induces high lymphocyte infiltration in the liver, whereas none of the other treatments did (Figures 6A–6C). Another sign of systemic inflammation is splenomegaly, which was only triggered by 4-1BB agonistic antibody (Figure 6D). Hematoxylin-eosin staining was also used to assess T cell infiltration and signs of liver damage, and again no apparent sign of inflammation or tissue alteration was detected (Figure 6E).

## Discussion

Cancer immunotherapy is based on the idea of inducing or engrafting a specific immune response against tumor cells. Taking advantage of the exquisite specificity of the immune system to recognize and destroy non-self-antigens, there is a great interest in the development of personalized vaccines aimed at identifying the best neoantigens derived from somatic tumor mutations in each patient. This approach has shown very promising results in cancer patients, but it is still technically cumbersome and far from being broadly available to everyone, not to mention that there might still be patients with lower mutation rates that do not yield a good repertoire of immunogenic neoantigens. While working with Eli Gilboa, we proposed a radically different approach to amplify the antigen repertoire by inhibiting the RNA surveillance pathway NMD, which eliminates potential strong neoantigens derived from aberrant transcripts containing premature stop codons.^14^

NMD inhibition in the tumor may also have potentially opposite effects.^32^ These apparent contradictory effects probably hold the initial interest to develop therapeutic drugs to block NMD in cancer. Nonetheless, NMD inhibition is a key ally in cancer immunotherapy^14^^,^^23^^,^^33^ and chemotherapy.^34^ Additionally, recent studies have underscored the existence of NMD dependencies as a predictor of tumor antigenicity in different cancer types.^17^^,^^33^^,^^35^^,^^36^ Outside the context of cancer immunotherapy, there are other studies in which NMD blockade can be protumorigenic.^32^ Quite likely, the yin-yang role of NMD in cancer might depend on the time frame of the NMD blockade as it occurs with other immune targets (e.g., TGF-β, CTLA-4).^37^^,^^38^ Time-sustained NMD inhibition can also lead to non-tumor-tissue inflammation probably by the expression of universal cryptic antigens or by the induction of other stress immune cycles.^39^^,^^40^ To the best of our knowledge, NMD mutation as potential tumor driver has been reported only in two different cancer entities: (1) inflammatory myofibroblastic tumors,^41^ a peculiar type of cancer characterized by massive leukocyte infiltration with good prognosis, and (2) pancreatic adenosquamous carcinoma,^42^ in which actual NMD dependence has recently been challenged.^43^

To reduce undesirable inflammatory associated side effects, it is important to design a therapeutic agent that can inhibit NMD transiently and mainly in the tumor tissue. RNAi targeting has been clinically successfully thanks to GalNAc RNAi conjugates.^43^ Another viable option for RNAi delivery is to use a carrier aptamer RNAi targeting platform.^22^ We choose to use a DNA aptamer AS1411 that is well validated and tested in different clinical trials for its cytotoxic antitumor response.^25^ The size of the aptamer allows the chemical synthesis of the oligo together with RNA antisense sequence as a single chimeric oligonucleotide that is later hybridized with the passenger strand of SMG1 siRNA. The aptamer binds to different tumor cell lines and allows for the free uptake of siRNA cargo to inhibit the SMG1 mRNA, leading to NMD activity downregulation stabilizing encoded antigens.

We describe a broadly clinically translational aptamer RNAi AsiC to inhibit SMG1 in a wide range of tumor types. Downregulation of SMG1 by aptamer targeting AsiCs impedes NMD activity, possibly leading to the stabilization of neoantigens that are constantly eliminated via NMD. This increase in immunogenicity triggers the homing of T-reactive lymphocytes to the tumor, hence inflaming the tumor milieu. Mass spectrometry analysis is required to determine how the antigen repertoire is reshaped by NMD inhibition.^19^ Herein, we use a simpler indirect method to address this possibility by expressing an artificial determinant antigen that contains a PTC under the NMD control. Thus, we confirm that NMD can regulate antigen expression, but future mass spectrometry MHC-ligandome studies will finally corroborate if this is the case also for endogenous neoantigens.

It is possible that private and/or universal neoantigens remain elusive to the immune system by the constitutive activity of NMD. The importance of each type of neoantigen (private or universal) in the final outcome of the antitumor immune response upon NMD inhibition will probably depend on each type of tumor and needs to be elucidated in future experiments. Highly mutated tumors, as is the case of high-level microsatellite instability (MSI-H), will likely be encoding many private neoantigens that are silenced by NMD,^44^ in which inhibition may improve overall tumor antigenicity. MSI-H tumors usually respond well to the ICB therapy thanks to high basal neoantigen load,^44^ but there are still a few non-responding patients or others that develop ICB resistance. Therefore, NMD inhibition could be a choice to raise the rates of response to ICB in these types of tumors. A more challenging situation hinges on tumors with low mutation rates (low antigenic tumors); in this scenario it will be desirable to upregulate universal cryptic neoantigens silenced by NMD. A recent study by Liberman’s team^23^ has shown that NMD inhibition with an Upf2 AsiC in combination with Parp1, Mcl1, and CD47 tumor target inhibition leads to disease control in Balb neut Erb2ΔEx16 mice. These transgenic mice develop a very aggressive spontaneous breast tumor that is triggered by a single oncogene activation (Erb2 with the in-frame-deletion of exon 16, which elicits constitute activation of Erb2). The number of other accumulated mutations (potential neoantigens) in a tumor of these transgenic mice is quite limited as the tumor progresses very fast, driven only by the Erb2 oncogene. Thus, in this situation the antitumor response induced by NMD inhibition could be justified by allowing the expression of universal cryptic antigen derived from mRNA transcription byproducts targeted by NMD. Apart from the increased amount and better quality of tumor neoantigens, there might be other mechanisms associated with NMD inhibition that contribute to enhancing the tumor immune cell infiltration as NMD controls cell-stress transcription wires. Further studies will likely address this possibility as well.

Vaccine efficacy to trigger an immune response depends on the adequacy of a chosen antigen as well as combination with the right adjuvant. Adjuvants are molecules that alert the immune system to respond; usually this type of signal accounts for microbial products known as pathogen-associated molecular patterns and for intracellular molecules released during cell death known as damage-associated molecular patterns (DAMPS). Radiotherapy and some chemotherapy drugs display an adjuvant effect in cancer immunotherapy by releasing DAMPS and favoring the antitumor immune response.^45^^,^^46^ The caveat with radiotherapy or chemotherapy is that their cytotoxic effects can be exerted also in immune cells hampering the potential antitumor immune response. Tumor-targeting cytotoxic agents might spare the immune cells allowing the full display of the antitumor immune response. Furthermore, with AS1411-SMG1-AsiC, if the cytotoxic targeting drug releases a therapeutic cargo (SMG1 RNAi) that amplifies the antigen load, we have the optimal platform to create an endogenous immune response, as shown by the high immune infiltrate triggered by the AS1411-SMG1 AsiC treatment.

The high immune infiltration induced by AS1411-SMG1 AsiC creates the optimal conditions to improve the rate of response to ICB. We chose the combination of anti-CTLA-4/PD-1 antibodies as the gold standard immunotherapy treatment based on positive results in clinical trials.^47^, ^48^, ^49^ B16/F10 melanoma cells are more sensitive to the cytotoxic effect of AS1411 with lower IC_50_ than 4T1 (Figure 1C), and therefore it is expected that its combination with SMG1 inhibition elicits a stronger antitumor response compared with the 4T1 model (Figure 5). Based on that, we decided to use the 4T1 model to evaluate the additive effect of ICB therapy. The AS1411-SMG1 AsiC and ICB combo shows a significant reduction in tumor progression in the 4T1 tumor model (Figure 5D). Of note, each treatment separately in 4T1 model (ICB or AS1411-SMG1 AsiC) shows limited antitumor effect, indicating that the target inhibition of NMD might be a useful therapeutic tool for refractory tumors that do not response to ICB*.*

Despite the intrinsic limitations of mouse-based toxicology studies in resembling the toxic effects of many drugs in humans, it is important to underscore that they recapitulate well the immune-related adverse events (irAEs) observed with various drugs used in cancer immunotherapy such as the anti-4-1BB agonistic antibody.^50^ We have to further evaluate the possible toxic effect of AS1411-SMG1 AsiC compared with other immunotherapy regimes. Even though we have not performed dose escalation studies to assess the maximal tolerable dose, we did not observe major irAEs under the therapeutic conditions that elicit an antitumor response.

## Materials and methods

### Animals

C57/BL6 and Balb/c mice were purchased from Envigo. All mice were housed in Center for Applied Medical Research (CIMA) animal facility (CIMA, Pamplona, Spain). Animal experiments were conducted using 6- to 8-week-old C57/BL6 or Balb/c female mice. OT-I mice were bred in our facilities (CIMA, Pamplona, Spain). Animal studies were approved by the Animal Ethical Committee of the University of Navarra in the veterinary facilities of the CIMA following the institutional as well as national laws and ethical guidelines for experimental animal care.

### Cell lines and culture conditions

ARST, AXBI, and Panc02 were a kind gift from Dr. I. Melero (CIMA, Pamplona, Spain). 4T1 cells were provided by Dr. F. Lecanda (CIMA, Pamplona, Spain) and B16/F10 by S. Hervás-Stubbs (CIMA, Pamplona, Spain). Cell lines were cultured in RPMI-1640 medium (4T1, CT26, ARST, and AXBI), Dulbecco’s modified Eagle’s medium (DMEM) (B16 and Panc02) (all from Gibco) supplemented with 8%–10% heat-inactivated FCS, 100 U/ml penicillin, and 100 μg/mL penicillin/streptomycin. OT-I splenocytes medium consisted of RPMI that was additionally supplemented with 1 mM sodium pyruvate (all from Gibco), 0.05 mM β-mercaptoethanol (Sigma), 1 mM HEPES, and 1X minimal essential medium (MEM) non-essential amino acids (all from Gibco). All cell lines and assay cultures were maintained at 37°C and 5% CO_2_. All cells were mycoplasma-free and tested regularly using *MycoAlert* PLUS Mycoplasma Detection Kit (Lonza).

### AS1411 AsiCs

Characterization of AS1411-siRNAs conjugates: AS1411 ssDNA aptamer(dGdGdTdGdGdTdGdGdTdGdGdTdTdGdTdGdGdTdGdGdTdGdGdTdGdG), extended at the 3′ end with SMG1 siRNA (rG/i2FC//i2FC/rA/i2FC//i2FC/rArArArGrA/i2FC/rA/i2FU/rGrArGrGrArArA) or a control siRNA (/52FC/rArArG/i2FC//i2FU/rGrA/i2FC//i2FC/rC/i2FU/rGrArArG/i2FU/rUrC) were purchased from Integrated DNA Technologies (IDT) with the indicated 5-fluorouracil modification in pyrimidines. Complementary oligos of both RNA were also supplied by IDT: SMG1 (rUrUrUrCrCrUrCrArUrGrUrCrUrUrUrGrGrUrGrGrC) and control (rGrArArCrUrUrCrArGrGrGrUrCrArGrCrUrUrG). Antisense (guide strand) siRNA were hybridized in annealing buffer (NaCl 0.15 M; EDTA 0.01 M; Tris-Cl pH 8.8; adjusted to final pH = 7.5) in a thermocycler starting at 65°C and allowed to cool to 37°C. Correct hybridization of the AS1411 AsiCs was checked in a 15% acrylamide SDS-PAGE (Figure S1A). Expected molecular weight was assessed with RNA Marker Low (Abnova).

For *in vitro* validation, we first validated the AsiCs binding to murine cells by flow cytometry. The guide strand of the SMG1 siRNA was chemically synthesized with AlexaFluor on its 5′-end and hybridized with AS1411-SMG1 or ssDNA Scramble (dTdTdTdCdCdTdCdCdTdCdCdTdCdCdTdTdCdTdCdCdTdCdCdTdCdCdTdC)-SMG1 AsiCs (all from IDT). Both AsiCs presented 5-fluorouracil modifications in their pyrimidines. Cells were incubated with the AlexaFluor-tagged AsiCs for 30 min at 37°C in PBS, washed twice, and analyzed in a CytoFLEX flow cytometer (Beckman Coulter). Next, we checked silencing in murine cells by transfecting 100 pmol AS1411-AsiCs SMG1 and control with Lipofectamine 2000 (Invitrogen) following manufacturer’s instructions. For free uptake assays, 3 x 10^5^ CT26 cells were seeded in a six-well plate. The day after they were treated with 250 pmol of AS1411 AsiCs diluted in 500 μL of OptiMEM medium (Gibco) for 2 h. Then 1.5 mL of complemented 1640-RPMI medium (10% FBS, 1% L-glutamine, 1% penicillin/streptomycin, all from Gibco) was added. The same procedure was repeated the following day. RNA was isolated using QIAGEN mini kit. SMG1 mRNA levels were measured by qRT-PCR. Primers used were the following: (forward: TGTGACCAGCCCTGAGTTTAC; reverse: CGAGACTCATCAGAGTACGACAT). HPRT served as housekeeping control (forward: TCCTCCTCAGACCGCTTTT; reverse: CCTGGTTCATCATCGCTAATC). For western blotting, Panc02 cells were treated following the same schedule as in the qRT-PCR experiment. Tumor cells were homogenized in lysis buffer: PBS containing 10% Triton X-100 (Sigma) with cOmplete Protease Inhibitor Cocktail (Roche) for 30 min in ice. Samples were then centrifuged for 15 min at 10,000 rpm at 4°C. Protein concentration in the resulting supernatants was quantified using Protein Assay Dye Reagent Concentrate (BioRad) diluted in deionized water. Equal amounts of lysates were fractionated by BioRad mini-PROTEAN TGX 4 15% gels (BioRad) and electrotransferred to 0.45-μm pore size nitrocellulose membranes (BioRad). After blocking with TBS (BioRad)/0.1% Tween (Sigma)-20/5% milk, the membranes were probed with rabbit anti-mouse SMG1 (Cell Signaling; 1:1,000; clone Q25) and rabbit anti-mouse β-Actin (Cell Signaling; 1:2,000; clone 13E5) o/n in agitation at 4°C. HRP-linked anti-rabbit antibody (Cell Signaling; 1:5,000) was used as secondary antibody. Protein bands were detected by chemiluminescence using Amersham ECL Western Blotting Detection Reagents (GE Healthcare) in a ChemiDoc device (BioRad).

psiCHECK assay: 5 x 10^3^ Panc02 or B16/F10 cells per well were seeded in a flat bottom 96-well plate. Cells were treated twice with 100 pmol of AS1411 AsiCs (control or SMG1) at 24 h and 48 h for free uptake treatment in 100 μL of OptiMEM medium (Gibco) for 2 h, and then 100 μL of complemented DMEM was added. The same amount of AsiCs was transfected with Lipofectamine 2000 (Invitrogen) following siRNA manufacturer’s protocol as control of the assay. Renilla and Firefly signals were measured with Dual-Glo Luciferase Assay System (Promega).

For OT-I activation by NMD-inhibited B16 expressing SIINFEKL-β-Globin-PTC39 cassette (Figure S2), 1.5 x 10^5^ B16-OVA-PTC39 were seeded in a six-well plate per well. Cells were incubated with AS1411 AsiCs as described previously in this materials and methods section. Treated cells were collected and 5 x 10^4^ B16 were co-cultured in a U-bottom 96-well plate with 5 x 10^5^ OT-I splenocytes. IFN-γ production was detected using BD OptEIA Mouse IFN-γ ELISA Set (Beckton Dickinson).

### AS1411 MTS

50 B16/F10, 4T1, or MDA-MB-231 cells were plated in a flat bottom p96-well plate (BD) in 100 μL of complete medium. The next day, cells were treated with AS1411 at concentrations of 100–0.4 μM in 1:2 serial dilutions for 6 days when clear growth differences were spotted. AS1411 was diluted in complete medium and 100 μL per well was added. Vehicle-treated cells were used as negative control. At day 6, medium with AS1411 was removed, and 100 μL of complete medium mixed with 20 μL of CellTiter 96 AQueous One Solution Cell Proliferation Assay (Promega) was added per well and incubated for 1 h. Absorbance was measured at 490 nm in a SPECTROstar Nano (BMG Labtech) spectrophotometer. A four-parameter, non-linear regression test was performed to calculate IC_50_ values using GraphPad Prism 7.0.

### AS1411-AsiC antitumor therapy experiments

For intratumoral administration of AS1411 AsiCs in B16/F10 melanoma model, 1.5 x 10^5^ B16/F10 melanoma tumor cells were implanted in the right flank of 6- to 8-week-old C57/BL6 female mice. 300 pmol of AS1411-SMG1 or control was administered intratumorally at days 1, 2, 3, 10, 14, and 15 post-tumor inoculation. At day 16, animals were sacrificed to analyze tumor infiltrate by flow cytometry and to measure tumor weights.

For intratumoral administration of AS1411 AsiCs in CT26 colon carcinoma model, 3 x 10^5^ CT26 colon carcinoma cells were injected in 6- to 8-week-old Balb/c female mice. 300 pmol of AS1411 AsiCs (SMG1 or control) was administered at days 4, 6, 8, 11, 14, and 16 post-tumor inoculation intratumorally. Mice were sacrificed at day 20, and tumor weight was measured.

For systemic administration in B16/F10 melanoma model, 1.5 x 10^5^ B16/F10 were implanted in the right flank of 6- to 8-week-old C57/BL6 female mice. 300 pmol of AS1411-SMG1 or control was administered intravenously at days 1, 2, 3, 10, 14, and 15 post-tumor inoculation.

For systemic administration and combination with anti-CTLA-4 and PD-1 antibodies cancer breast 4T1 model, 5 × 10^4^ breast cancer tumor cells were implanted in the right flank of 6- to 8-week-old Balb/c mice. 300 pmol of AS1411 AsiCs SMG1 or control was injected systemically at days 1, 2, 3, 10, 14, and 15. 100 μg CTLA-4 (clone 9H10) and PD-1 (clone rmp1-14) or 200 μg of Rat IgG2a isotype control (clone 2A3) (all from Bio X Cell) was intraperitoneally administered at days 1, 3, and 7.

Tumor volume in all experiments was measured using a caliper three times per week at the indicated time points represented in the figures. Plotted values correspond to volumes that were calculated using the following formula: tumor volume = [length × (width)^2^]/2.

### Flow cytometry

For tumor infiltrate studies by flow cytometry, tumors were resected on day 16 after implantation. Each tumor was placed in a 100-mm Petri dish (Greiner Bio-One) and digested with 5 mL of RPMI medium containing collagenase D (400 U/ml) and 50 μg/mL DNase I (both from Roche) for 30 min at 37°C. After incubation, 100 μL of 0.5 M EDTA (Invitrogen) was added to the tumors to stop the reaction. Tumor samples were smashed and filtered through a 40-μm nylon cell strainer (Falcon) to a 50-mL centrifuge conical tube (Corning). Cells were pelleted at 1,700 rpm for 5 min RT. Supernatants were discarded, and erythrocytes were lysed using 1 mL of ACK lysis buffer (Gibco) for 1 min on agitation. PBS-EDTA (2 mM) was added up to 50 mL to neutralize the lysis, and cells were spun down again at 1,700 rpm for 5 min. Each pellet was resuspended in PBS and spun down in a V-bottom 96-well plate (Thermo Fisher Scientific) at 1,800 rpm for 1 min. Cells were resuspended in 80 μL of Zombie Aqua mix (BioLegend) diluted 1:500 and incubated for 15 min at RT protected from light. Then cells were stained with the following antibody mix: CD45-APC-Cy7 (Clone 30-F11), CD8-APC (Clone 53.67), CD4-BV510 (Clone GK1.5), and CD3e-BV421 (Clone 145-2C11) (all from BioLegend) for 20 min at RT protected from light. After this stage, cells were washed twice, and FOXP3-PE (Clone FJK-16s; Invitrogen) intracellular staining was performed using eBioscience Foxp3/transcription factor buffer set (Thermo Fisher Scientific) following manufacturer’s instructions.

For aptamer staining, 3 x 10^5^ cells were resuspended in 50 μL of PBS per sample in a V-bottom plate (Thermo Fisher Scientific) after two washes in PBS. Cells were stained with 1 pmol of AS1411-Biotin or Scramble-Biotin (Biotin-GGTTGATGGTATGGATACCCTGG) (both purchased from Sigma) and 0.1 μg of Streptavidin-PE (BioLegend) for 30 min at 37°C protected from light. Samples were washed twice in PBS and analyzed in a CytoFLEX flow cytometer (Beckman Coulter) and analyzed in FlowJo X (FlowJo). See Figure S3for gating strategy.

For hepatotoxicity assays liver samples were homogenized and filtered through a 40-μm nylon cell strainer (Falcon) to a 50-mL centrifuge conical tube (Corning). Cells were pelleted at 1,700 rpm for 5 min RT. Supernatants were discarded, and erythrocytes were lysed using 5 mL of ACK lysis buffer for 2.5 min with agitation. PBS-EDTA was added up to 50 mL to neutralize the lysis, and cells were spun down again at 1,700 rpm for 5 min. The pellet was resuspended in PBS and spun down after transfer to a V-bottom 96-well plate (Thermo Fisher Scientific) at 1,800 rpm for 1 min. Cells were stained with CD3-BV421, CD8-APC, or CD4-BV421 antibodies (the same as the ones used for tumor infiltrate studies).

### Immunohistochemistry

Paraffin sections (3 μm thick) were cut, dewaxed, and hydrated. Antigen retrieval was performed for 30 min at 95°C in 0.01 M Tris-1 mM EDTA solution (pH = 9) in a Pascal pressure chamber (DAKO S2800). Slides were allowed to cool for 20 min; then endogenous peroxidase was blocked with 3% H_2_O_2_ in deionized water for 12 min, and sections were washed in TBS-0.05% Tween 20 (TBS-T). Sections were incubated overnight at 4°C with CD3 antibody (Clone SP7) (Thermo Fisher Scientific). After rinsing in TBS-T, the sections were incubated with goat anti-rabbit labeled polymer EnVision + System (Dako) for 30 min at RT, and peroxidase activity was revealed using DAB+ (Dako). Finally, sections were lightly counterstained with Harris hematoxylin, dehydrated, and cover-slipped with Eukitt (Labolan). Samples were scanned in an Aperio CS2 (Leica Biosystems) using a 20X lens.

### Toxicity assays

Tumor-free 6- to 8-week-old female C57/BL6 mice were injected systemically with 300 pmol of AS141-SMG1 AsiC on days 1, 2, 3, 10, 14, and 15 following the same schedule as for antitumor experiments. 100 μg of CTLA-4 and 100 μg of PD-1 or 200 μg of 4-1BB (Clone 3H3; Bio X Cell) antibodies were intraperitoneally administered at days 1, 3, and 7. On day 20, mice were sacrificed, and spleen and liver were extracted. Spleen samples were weighed to study splenomegaly. Liver samples were processed for flow cytometry (see flow cytometry section of this materials and methods) and additionally used for paraffin embedding. 5-μm slices were stained with hematoxylin and eosin staining to detect tissue damage and immune cell infiltration.

### Quantification and statistical analysis

Data were processed using GraphPad Prism 7.0, and all figures show mean ± SEM. Flow cytometry analysis was performed with FlowJo 10. Error bars represent SEM in all plots. One-way ANOVA followed by post-hoc Bonferroni test was performed to analyze statistical differences between independent groups. For *in vivo* experiments, treatment effect was determined by using two-way ANOVA with Bonferroni test. Statistical significance is considered at p < 0.05. When differences are statistically significant, the significance is represented with asterisks (^∗^) according the following values: p < 0.05(^∗^), p < 0.01(^∗∗^), p < 0.001(^∗∗∗^), and p < 0.0001 (^∗∗∗∗^). For *in vivo* experiments with several time points, asterisks show the significance of the final time point.

## Data Availability

Data and materials would be available upon request.

## Supplemental information

Document S1. Figures S1–S 3

Document S2. Article plus supplemental information
