# Supplementary material for: A pan-tumor-siRNA aptamer chimera to block nonsense-mediated mRNA decay inflames and suppresses tumor progression
Source: Mol Ther Nucleic Acids. 2022 Jul 20;29:413–25. doi: 10.1016/j.omtn.2022.07.017 (PMC9379514; doi:10.1016/j.omtn.2022.07.017)
Supplement: Document S1. Figures S1–S — 3 [file mmc1.pdf]

**OMTN, Volume *29***

## Supplemental information

# A pan-tumor-siRNA aptamer chimera to block nonsense-mediated mRNA decay inflames and suppresses tumor progression

**Daniel Meraviglia-Crivelli, Helena Villanueva, Ashwathi Puravankara Menon, Angelina Zheleva, Beatriz Moreno, María Villalba-Esparza, and Fernando Pastor**

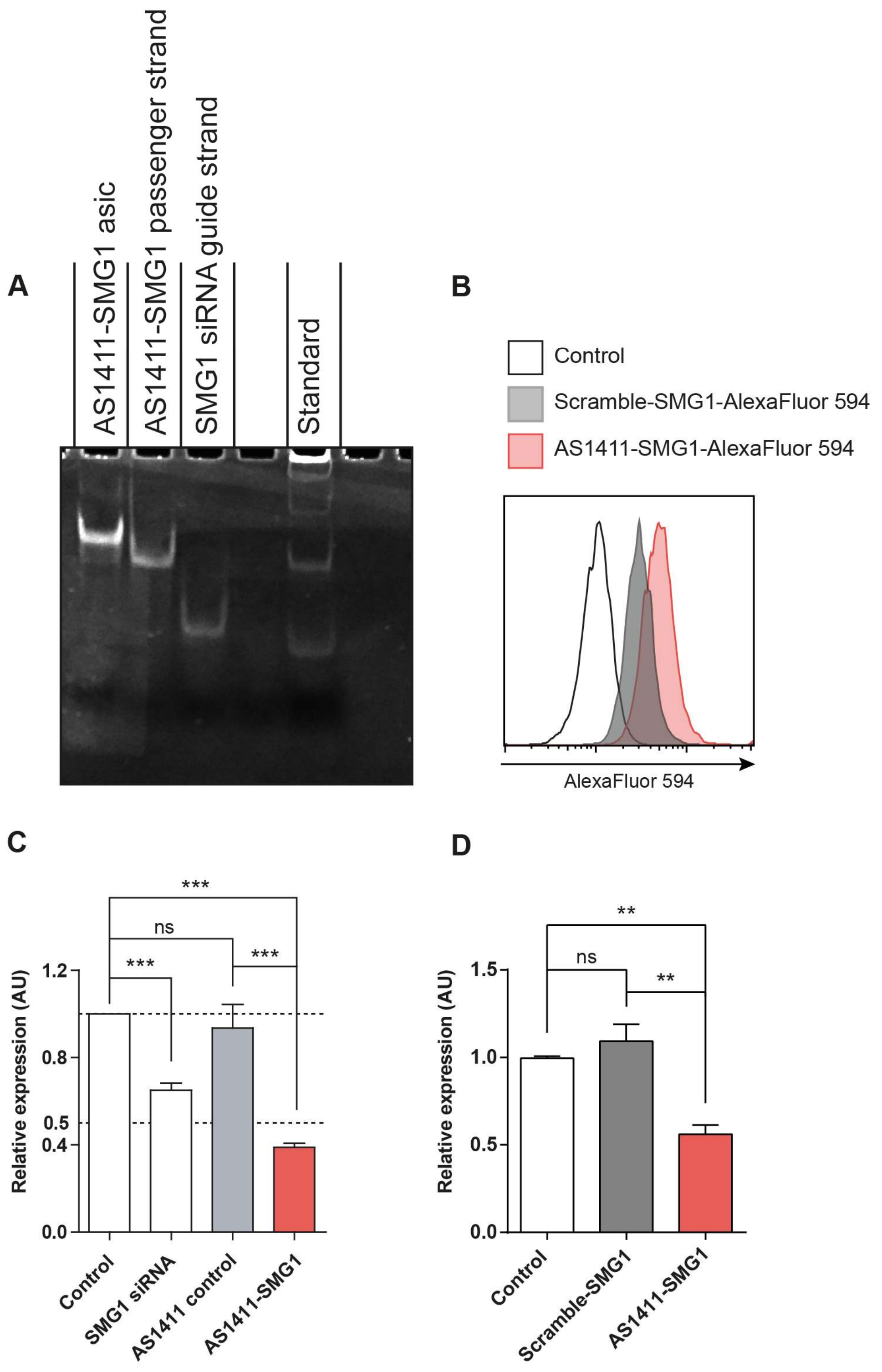


**Fig S1.** Validation of AS1411 AsiC *in vitro.* A) Characterization of AS1411-SMG1 AsiC by PAGE after chimera production to assess correct hybridization. Unhybridized passenger and guide strands are also shown. B) *In vitro* binding of the AS1411-SMG1 AsiC to B16-F10 cell line measured by flow cytometry. C) AS1411-SMG1 AsiC preserves RNAi activity. CT26 mouse colon cancer cell line was transfected with SMG1 or control aptamer construct. Cells were harvested after 24 h of incubation and SMG1 silencing was measured by qRT-PCR. Technical triplicates. n = 1. A Scramble-SMG1 chimera was used as control. D) Scramble-SMG1 AsiC does not induce target silencing *in vitro* in CT26 cell line when added to cell medium. AS1411-SMG1 AsiC was used as positive control. SMG1 levels were measured by qRT-PCR.

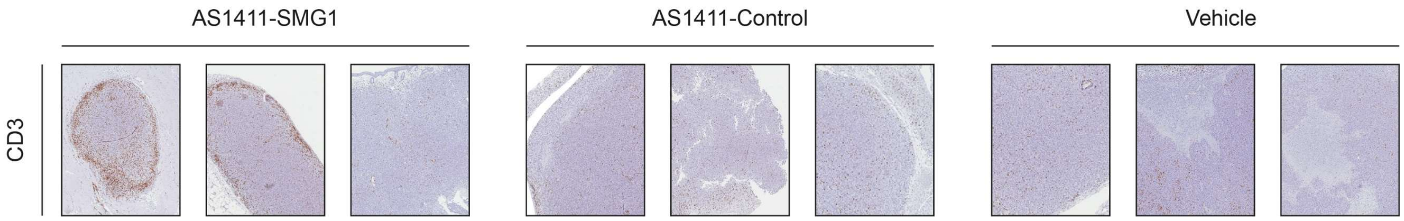


**Fig S2.** Immunohistochemistry of CT26 tumors after AS1411 AsiCs treatment. CT26 cells were implanted into the right flank of Balb/c mice and treated with 6 doses of AS1411 AsiCs or vehicle administered via intratumoral injection. Tumors were resected on day 20 and embedded in paraffin. Cuts were mono-stained with a CD3 antibody. n = 3.

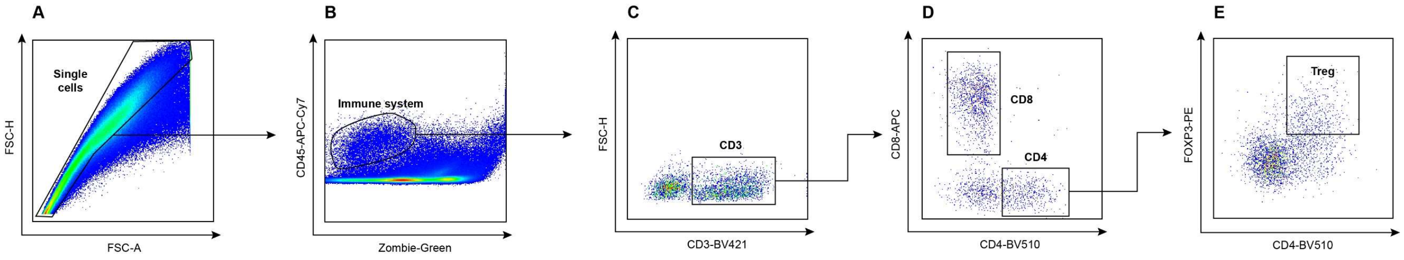


**Fig S3.** Leukocyte gating strategy for phenotypic characterization of B16/F10 tumor infiltrate after AS1411 AsiCs treatment. After tumor homogenization and red blood cell lysis samples were stained with labelled antibodies (see Materials and Methods section). Expression data was collected by flow cytometry and analyzed using FlowJo. Statistical analyisis was performed with GraphPad Prism 7.0. A) Single cells were gated based on their forward scatter height and area. B) Immune infiltrate was identified by Zombie Green negative (live population) and CD45+ cells within the single cells group. C) CD3+ cells were tagged as the lymphoid cluster in which D) CD8+ and CD4+ T cells were identified. E) FOXP3+ cells were gated within the CD4+ population.
